# Hijacking Transposable Elements for Saturation Mutagenesis in Fungi

**DOI:** 10.3389/ffunb.2021.633876

**Published:** 2021-04-13

**Authors:** Sanne Schrevens, Dominique Sanglard

**Affiliations:** Institute of Microbiology, University of Lausanne and Lausanne University Hospital, Lausanne, Switzerland

**Keywords:** transposons, mutagenesis, fungi, *Candida*, fungal pathogens, yeast

## Abstract

Transposable elements are present in almost all known genomes, these endogenous transposons have recently been referred to as the mobilome. They are now increasingly used in research in order to make extensive mutant libraries in different organisms. Fungi are an essential part of our lives on earth, they influence the availability of our food and they live inside our own bodies both as commensals and pathogenic organisms. Only few fungal species have been studied extensively, mainly due to the lack of appropriate molecular genetic tools. The use of transposon insertion libraries can however help to rapidly advance our knowledge of (conditional) essential genes, compensatory mutations and drug target identification in fungi. Here we give an overview of some recent developments in the use of different transposons for saturation mutagenesis in different fungi.

## Introduction

All known genomes harbor transposable elements (TEs). These elements are genetic units that can move throughout the genome. Two main types of transposons exist: the retrotransposons (Class I), using a copy-paste mechanism and the DNA transposons, using a cut-paste mechanism (Class II) (Wicker et al., 2007; Kojima, 2020). In fungi, class I transposons have mainly been studied in the yeast *Saccharomyces cerevisiae*. Genomic copies of these retrotransposon sequences are transcribed and partly translated allowing the formation of virus-like capsids in which Ty RNAs are reverse transcribed into cDNA and imported into the nucleus for their integration into the host genome. In the case of the *S. cerevisiae* Ty elements, a transposition event results in the presence of both the original transposon sequence and the new copy in the genome (Curcio et al., 2015). Contrastingly, class II transposons are first excised from the genome and this excised DNA fragment is inserted in another genomic location with no direct increase in copy number. The distribution of TEs in the genome reflects the balance between their excision and insertion (Levin and Moran, 2011; Huang et al., 2012). Despite being poorly represented in Saccharomycotina, a number of class II transposable elements are present in various fungi (Castanera et al., 2016; Muszewska et al., 2017, 2019).

Most mobile elements are non-randomly distributed in genomes and have insertion preferences. This target site selection can depend on sequence preferences, interaction with host proteins (Zayed et al., 2003) and DNA accessibility (chromatin structure) (Gangadharan et al., 2010). *S. cerevisisae* Ty retrotransposons, for example, integrate in non-coding DNA. Ty1-4 all target different sequences upstream of RNA polymerase III transcribed genes, while Ty5 prefers integrating in the silent mating locus and the telomeres (Bonnet and Lesage, 2021). For class II transposons, target sequences include the TA dinucleotide for Mariner transposons and the TTAA target site for piggyBac (Mutumwinka et al., 2018; Zhu et al., 2018a). The Ac/Ds element from maize on the other hand, does not have a known target sequence (Michel et al., 2017).

Several fungal species are used as model organisms in research and collections of molecular tools have been designed to help their genetic manipulation (Hoffman et al., 2015; Alexander, 2018). However, such tools and especially engineered collections are usually only available in so-called laboratory strains, which are sometimes too distant from industrially, agriculturally, or clinically relevant isolates or species for a direct transfer of knowledge and molecular tools. Deletion mutant collections usually exhibit one complete deletion of each non-essential gene. Transposons could be helpful to create mutant collections by multiple insertions within and in the proximity of genes, and as such contain much more information than a classic deletion collection. Since transposon mutant collections are characterized in bulk, conditional fitness advantages or disadvantages conferred by insertions in a gene can readily be detected (Zhu et al., 2018b). Furthermore, because transposon mutant libraries enable genome-wide functional genomics exploration in any background, we anticipate that this method will greatly accelerate functional and genetic network-related annotation of genomes.

Additionally, as insertion into non-coding regulatory sequences, such as promoter regions, affects gene expression, transposon libraries enable selection of expression mutants (Patterson et al., 2018). Finally, the higher resolution resulting from a differential effect caused by insertions at different positions within the same gene in some cases enables to define essentiality at a protein domain level (Michel et al., 2017).

## Endogenous Transposons in *Saccharomyces cerevisiae* and Other *Fungi*

In the model yeast *Saccharomyces cerevisiae*, several active endogenous retrotransposons have been identified, of which the Ty family is the best studied (Curcio et al., 2015; Rowley, 2017). Ty elements are long terminal repeat (LTR) transposons with a total length of 5918-bp, 334-bp of which consist out of the LTRs. This sequence contains two open reading frames (ORFs): *GAG*, the capsid, and *POL*, a protease, integrase and reverse transcriptase (Curcio et al., 2015). Even though these transposons can be found in yeast, they only account for 1.3–3.4% of total genomic DNA, which is significantly lower compared to other eukaryotic organisms (Carr et al., 2012). The Ty1 transposable element has an impact on the yeast genome in regulating the expression of adjacent genes, especially under stress conditions. Furthermore, it was shown to influence initiation and repair of double stranded DNA breaks, genome evolution, and aging (Todeschini et al., 2005; Chan and Kolodner, 2011; Maxwell et al., 2011; Aguilera and Garcia-Muse, 2012; VanHoute and Maxwell, 2014). In addition to regulating expression of adjacent genes, Ty elements also tightly regulate their own mobility. Therefore, they bind either with retrotransposon-encoded antisense RNA or dominant negative proteins, or to host transcription factors and other proteins, which regulates their responses to external stimuli (Morillon et al., 2002; Matsuda and Garfinkel, 2009; Bilanchone et al., 2015; Nishida et al., 2015; Saha et al., 2015).

However, most Ty elements avoid insertion into coding sequences and are strongly intertwined with the host, making them less useful for research purposes such as the construction of transposon insertion mutant libraries (Curcio, 2019). More in-depth research on the presence of different retrotransposons in different fungi can be found in Novikova et al. (2009) and Muszewska et al. (2011). Unlike in *S. cerevisiae* and *Schizosaccharomyces pombe*, where only LTR retrotransposons are present, the genome of *C. albicans* harbors mostly retrotransposons, yet some DNA transposons are present (Maxwell, 2020). Only very few class II transposons are present in *Saccharomycotina*, they are, however, present in other fungi, but are much less investigated compared to class I transposons. The knowledge on eukaryotic DNA transposons is mainly based on studies performed in other organisms, such as plants and insects. Currently there are several known superfamilies of DNA transposons, which all have different insertion sites and preferences. They include the hAT transposons, the Tc1/Mariner family transposons and PiggyBac transposons. All three of these superfamilies have members with activity in a wide range of host organisms (Wicker et al., 2007; Li et al., 2013b; Kojima, 2020). Using bioinformatics approaches, it was found that all these transposon superfamilies were present in fungi (Muszewska et al., 2017, 2019). The Tc1 superfamily is the most common transposable element family in fungi. The population of these transposable elements in a genome is a result of the balance between two factors: the activity of the transposon and the defenses protecting the host against invasion by such mobile elements. Most *Saccharomycetes* spp. contain <20 copies of transposable elements with an active transposase domain. Silent transposons, which are lacking an active transposase and therefore cannot transpose are more common (Muszewska et al., 2017).

## Transposons Used for Saturation Mutagenesis in Fungi

As endogenous transposable elements are not ideal for insertion library construction in fungi due to their coevolution with the host, transposons coming from plants or insects are usually used. Furthermore, hyperactive transposase enzymes have been developed for some heterologous transposons and several genetic studies based on heterologous class II transposons have been carried out in fungi (Table 1) (Subramanian et al., 2009; Du et al., 2011; Yusa, 2015).

**Table 1:** Overview of described transposon mutagenesis experiments in fungi.

**Transposon**	**Transposase**	**Selection**	**Species**	**Application**	**Library size**	**References**
MiniDs	AcTPase4xCa	•Excision: pADE2 •Insertion: *NAT1*	*C. albicans*	•Gene essentiality	±600,000 unique insertions	Segal et al., 2018
MiniDs	AcTPase4x	•Excision: *ADE2*	*S. cerevisiae*	•Gene essentiality •Genetic interaction •Drug target identification •Essential protein domains	284,162 unique insertions	Lazarow et al., 2012; Michel et al., 2017
Hermes	hyHermes^G366W^ TPase	•Insertion: NATMX	*S. cerevisiae*	•Genetic interaction	322,123 unique insertions	Gangadharan et al., 2010; Edskes et al., 2018
Hermes	WT Hermes TPase	•Insertion: *URA3*	*S. pombe*	•Gene essentiality •Genome architecture	360,402 unique insertions	Guo et al., 2013
Hermes	Codon optimized WT Hermes TPase	•Insertion: *LEU2*	*Y. lipolytica*	•Gene essentiality •Specific conditional essentiality	534,589 unique insertions	Patterson et al., 2018
Hermes	Hyperactive Hermes TPase	•Insertion: NATMX	*S. cerevisiae*	•Sequence specificity	178,607 unique insertions	Gangadharan et al., 2010
Hermes	Hyperactive Hermes TPase	•Insertion: *NAT*r	*C. glabrata*	•Gene essentiality •Drug target identification	513,123 unique insertions	Gangadharan et al., 2010; Gale et al., 2020
TcBuster	TcBuster TPase	•Insertion: *HIS4*	*K. phaffii*	•Gene essentiality •Specific conditional essentiality	113,061 unique insertions	Zhu et al., 2018a
Sleeping beauty	Sleeping beauty TPase	•Insertion: *HIS4*	*K. phaffii*	•Gene essentiality •Specific conditional essentiality	79,296 unique insertions	Zhu et al., 2018a
PiggyBac	hyPBaseCa	•Insertion: *URA3*	*C. albicans*	•Gene essentiality •Drug target identification	191,490 uniqe insertions	Gao et al., 2018
PiggyBac	PBase[SV40-neo]	•Insertion: *URA4*	*S. pombe*	•Genetic interaction •Drug target identification	33,218 unique insertions	Li et al., 2011
PiggyBac	PBase	•Excision: *ScARG4* •Insertion: *URA3*	*K. phaffii*	•Specific conditional essentiality	Only selected mutants were tested	Zhu et al., 2018b
PiggyBac	hyPBase	•Insertion: *HIS3*	*S. cerevisiae*	•Genetic interaction	Only selected mutants were tested	Yusa et al., 2011; Mutumwinka et al., 2018

**Figure 1:**
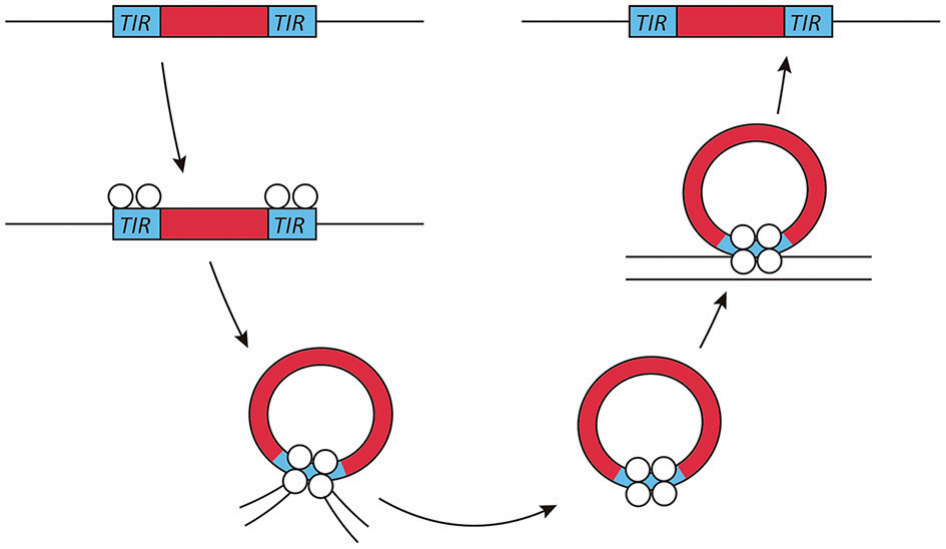
Transposon mechanism of class II DNA transposons. Class II or DNA transposons are first excised from the genome by a transposase. The terminal inverted repeats (TIRs) play an essential role in this process, as they probably lead to the temporary formation of a circular DNA fragment, which then reinserts into a different genomic location. This genome integration is probably helped by the integrase activity of the transposase.

Exogenous transposable elements used for insertional mutagenesis applications in fungi belong to the class II DNA transposon, since class I transposons are not as well-suited for insertional mutagenesis due to the presence of multiple insertions per genome. The DNA transposons move by a cut- and-paste mechanism. Active class II transposons usually have a single coding sequence encoding a transposase flanked by terminal inverted repeats (Figure 1). This transposase is required for both excision and insertion events. Most transposase enzymes contain a catalytic core sequence interspersed with arginine and lysine residues that are probably responsible for DNA interaction, as is the case in viral integrases (Keith et al., 2008).

### The hAt Transposon Superfamily

The first transposable element, discovered by McClintock in 1947, belongs to the Ac/Ds transposons, a part of the hAt transposon superfamily, which were mostly studied in plants (Du et al., 2011). The dissociator (Ds) element can excise from its locus in response to the activator (Ac) element, which encodes an active transposase flanked by terminally inverted repeats (TIRs) similar to those in the Ds element. Upon excision a small scar is left at the excision site and an 8-bp duplication is created at the target site upon transposition (Yan et al., 1999). The transposon prefers insertion in unique low copy sequences and tends to avoid highly repetitive DNA. Therefore, it preferentially inserts into or near coding sequences and tend to insert into closely linked regions. This feature makes Ac/Ds transposons highly suitable for insertion mutagenesis (Vollbrecht et al., 2010). However, Ac/Ds transposition frequencies are variable and in several species too low for large scale mutagenesis. Therefore, a hyperactive Ac transposase, discovered by Lazarow et al. (2012) is now widely used. The AcTPase4x carries a combination of four amino acid substitutions which result in a 100-fold increase in the excision frequency in yeast. The four mutations did slightly affect insertion preference for the insertion site: Typically, AcTPase4x prefers a slightly lower GC-content in its environment compared to wild type AcTPase (Lazarow et al., 2012). Noteworthy, both AcTPase and AcTPase4x form inactive protein aggregates when expressed a high levels. This is probably to protect the host from harmful transposition frequencies (Heinlein et al., 1994; Lazarow et al., 2012). So far, Ac/Ds was used successfully in the model yeast *S. cerevisiae* and haploid cells of the human fungal pathogen *Candida albicans* (Michel et al., 2017; Segal et al., 2018).

The hAT superfamily of transposons contains several other transposons that are used in fungi, such as Hermes, from the house fly *Musca domestica*. This mobile element exists of one single component, i.e., a transposase enzyme flanked by TIRs. Unlike its distantly related cousin, the Ac element from maize, it does integrate into a specific “nTnnnnAn” target sequence and also leads to a target site duplication of 8-bp. Up to now, Hermes has been used in the model yeasts *S. cerevisiae* and *Szichosaccharomyces pombe* and the human fungal pathogen *Candida glabrata* (Subramanian et al., 2009; Gangadharan et al., 2010; Edskes et al., 2018). The native Hermes TPase has been used in fungi as well as a hyperactive version (Subramanian et al., 2009; Gangadharan et al., 2010; Edskes et al., 2018). Next to Hermes, the TcBuster transposon from the flour beetle *Tribolium castaneum* was recently discovered in the hAT transposon family (Woodard et al., 2012). It differs from Hermes mainly in its DNA binding and insertion domain and in their insertion target site, which is “nnnTAnnn” for TcBuster (Arensburger et al., 2011). TcBuster was only used in *Komagataella phaffii*, previously classified as *Pichia pastoris* (Zhu et al., 2018a).

### The Mariner Superfamily

The Tc1/mariner transposons are the best-known superfamily of class II transposable elements. As in the hAT family, the transposon encodes a transposase enzyme, which is flanked by TIRs. Upon insertion into its target sequence, TA, this dinucleotide is duplicated (Dornan et al., 2015). In 1997 Ivics et al. (1997) genetically revived Sleeping Beauty an inactive Tc1/Mariner family transposon from fish. The authors used molecular phylogenetic data to guide the elimination of inactivating mutations within the gene encoding the transposase (Ivics et al., 1997). This transposon was used in *Komagataella phaffi* (Zhu et al., 2018a).

### The piggyBac Family

Third, the piggyBac transposon, originally from the cabbage looper moth *Trichoplusia ni*, forms its own family. It is currently the most widely used transposon system for genetic manipulations. PiggyBac-like elements are present in other moth species, frogs and bats, making it the first active transposon in mammals. This wide host range makes it very popular for several applications, such as determination of gene essentiality and drug target identification (Yusa, 2015; Gao et al., 2018). It is a 2475-bp long autonomous mobile element, containing TIRs and subterminal inverted repeats on either end. It contains a single open reading frame, which encodes the piggyBac transposase (Elick et al., 1996). PiggyBac shows very precise excision, leaving no excision scars. Furthermore, it is a unique eukaryotic transposon, as no DNA synthesis is required and excision occurs through hairpin formation, as described for specific bacterial transposons (Bischerour and Chalmers, 2007; Mitra et al., 2008). PiggyBac always inserts in a TTAA target-site, as this palindromic sequence is required for its insertion (Yusa, 2015). In addition, it shows a strong trend to hop locally, thus inserting close to the donor site, preferably in the same chromosome and in accessible chromatin structures (Li et al., 2013a). An optimized engineered non-autonomous piggyBac transposon system was first constructed and used in the silk worm *Bombyx mori* (Tamura et al., 2000). Further optimization was performed over the years to adapt the system to different species. Different markers, with a size of up to 10 kb, can be inserted between the TIRs of the transposon and, as such, can be mobilized without losing transposition efficiency (Yusa, 2015). A hyperactive transposase, hyPBase, was developed to increase transposition efficiency. Error prone PCR was used in order to generate a pool of mutant PBase DNA. Upon a subsequent screening in a *S. cerevisiae* transposition assay, several mutants with excision frequencies up to seven times higher compared to the wild type were identified. The hyPBase maintains the scarless transposon excision typical for piggyBac (Yusa et al., 2011). The piggyBac transposon has been used in the model organism *S. cerevisiae*, the industrial yeast *K. phaffii* and the human fungal pathogen *C. albicans* (Gao et al., 2018; Mutumwinka et al., 2018; Zhu et al., 2018b).

## Transposition Strategies Used in Saturation Mutagenesis in Fungi

Effective transposition in fungi requires expression of an active transposase, and a vector containing the transposon either inside a selectable gene or carrying a selection marker as a cargo, allowing selection of excision or insertion events, respectively. For the transposase, in general, three strategies are used: it can be expressed upon integration into the genome using an inducible promoter (Li et al., 2011; Gao et al., 2018; Mielich et al., 2018; Segal et al., 2018; Zhu et al., 2018a), using a plasmid-based inducible promoter (Gangadharan et al., 2010; Guo et al., 2013; Michel et al., 2017; Edskes et al., 2018; Mutumwinka et al., 2018; Zhu et al., 2018b; Gale et al., 2020) and using a plasmid-based constitutive overexpression system (Patterson et al., 2018). Ideally, an inducible promoter is used for control of the transposase and therefore for inducible transposition. However, since inducible promoters are often leaky, it is advisable to express the transposase from a plasmid that can be removed after transposition in order to create a library of stable transposon mutants.

**Figure 2:**
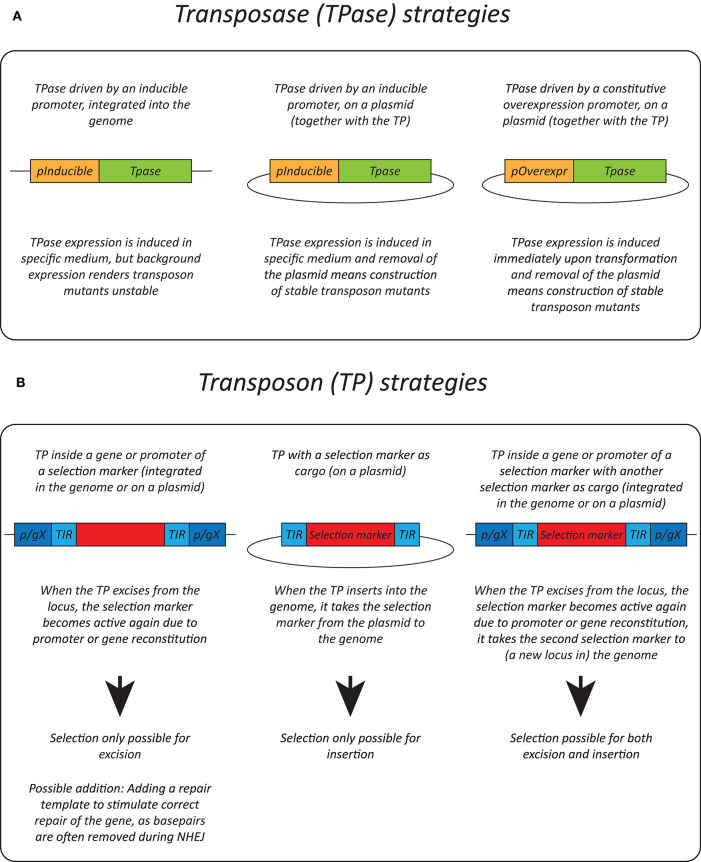
Transposition strategies in fungi. **(A)** Different strategies used for transposase (TPase) expression in yeasts. **(B)** Different selection methods used for transposon (TP) excision and insertion in yeasts. p/gX, promoter/gene X.

Different selection strategies were used for saturated transposon mutagenesis. First, the transposon can be inserted into a promoter or a gene rendering it inactive. Upon excision, the transcription of a functional gene is restored, enabling selection. For example, the MiniDs in *S. cerevisiae* was inserted into the *ADE2* gene, resulting in red colonies due to a defect in adenine biosynthesis. Upon transposition, the *ADE2* gene is repaired, resulting in white colonies (Michel et al., 2017). However, most transposons leave an excision scar, which makes it harder to use this selection method, as incorrect repair can affect selection. This can be solved by adding a functional gene template to repair the excision scar (Michel et al., 2019). A different selection method inserts the selection marker inside the transposon as a cargo. In this way, the marker is carried with the transposon to a genomic locus, as such selecting for insertion events. This method, however, can only be used when the transposon is added on a plasmid, as no selection for excision is present. This method depends on the transposons capability to carry a cargo (Gangadharan et al., 2010; Guo et al., 2013; Edskes et al., 2018; Gao et al., 2018; Mutumwinka et al., 2018; Patterson et al., 2018; Zhu et al., 2018a; Gale et al., 2020). Thirdly, it is also possible to combine the two methods, inserting the transposon in a selectable feature, as well as inserting a marker into the transposon as a cargo. This enables selection of transposon excision as well-insertion and makes it possible to insert the transposon into the genome at the start of the experiment, which is a necessity in fungi that do not support plasmids (Li et al., 2011; Mielich et al., 2018; Segal et al., 2018; Zhu et al., 2018b). Figure 2 gives a summary of strategies for transposase expression and transposon constructs for selection and Table 2 shows the pros and cons for each strategy.

**Table 2:** Transposon strategies in fungi.

**Transposase (TPase)**	**Pro**	**Con**	**References**
TPase driven by an inducible promoter, integrated into the genome	•Usable in most fungi	•Inducible promoters are often leaky and transposon mutants are unstable (the transposase can work on inserted transposons and excise them again)	Li et al., 2011; Gao et al., 2018; Mielich et al., 2018; Segal et al., 2018; Zhu et al., 2018a
TPase driven by an inducible promoter, on a plasmid	•Stable transposition upon removal of the plasmid	•Some fungi do not support plasmids	Gangadharan et al., 2010; Guo et al., 2013; Michel et al., 2017; Edskes et al., 2018; Mutumwinka et al., 2018; Zhu et al., 2018b; Gale et al., 2020
TPase driven by an overexpression promoter, on a plasmid	•Stable transposition upon removal of the plasmid	•Some fungi do not support plasmids •Transposition can only be controlled by removal of the plasmid	Patterson et al., 2018
**Transposon (TP)**	**Pro**	**Con**	
Inside a selectable feature (on a plasmid or in the genome)	•Transposition from the genome is possible in most fungi	•Some transposons leave excision scars, making selection less efficient	Michel et al., 2017
With a selection marker as cargo, on a plasmid	•Clear insertion selection upon removal of the plasmid	•Some fungi do not support plasmids	Gangadharan et al., 2010; Guo et al., 2013; Edskes et al., 2018; Gao et al., 2018; Mutumwinka et al., 2018; Patterson et al., 2018; Zhu et al., 2018a; Gale et al., 2020
Inside a gene or promoter of a selection marker with another selection marker as cargo, integrated in the genome or on a plasmid	•Transposition from the genome is possible in most fungi •Clear insertion selection	•Some transposons leave excision scars, making excision selection less efficient	Li et al., 2011; Mielich et al., 2018; Segal et al., 2018; Zhu et al., 2018b

## Applications of Transposon Mutagenesis in *Saccharomycotina*

### Investigating (Conditional) Gene Essentiality

In model yeasts such as *S. cerevisiae* and *S. pombe*, a lot of information is available on essential genes and even conditionally essential genes, which are only essential under certain growth conditions. This information was acquired using deletion collections, such as the yeast deletion collection in *S. cerevisiae* (http://www-sequence.stanford.edu/group/yeast_deletion_project/deletions3.html) (Mira et al., 2009). Making such collections is, however, very labor-intensive and molecular tools need to be available in the investigated species. Deletion collections can be used to identify conditionally essential genes by either screening every mutant separately in a high-throughput screen, or using competition assays, where pools of barcoded mutants are grown together and their relative presence is quantified. Transposon mutagenesis can be used to identify essential genes or conditionally essential genes if enough unique insertions are acquired and if a transposon system is used without specific target sites (Mira et al., 2009; Novo et al., 2013).

In the model yeast *S. cerevisiae*, Michel et al. (2017) succeeded in full functional mapping of the baker's yeast genome using saturated transposon mutagenesis. Seven libraries were constructed in different environmental conditions using a transposon inserted in the *ADE2* gene, either encoded on a plasmid or endogenously. More than 280,000 independent insertions were identified, with no sequence preferences and no large genomic regions without insertions. The Ac/Ds transposon did favor inter-nucleosomal DNA and pericentromeric regions in case of excision from a centromeric plasmid or targeted close to the excision site in case of excision from the endogenous *ADE2* locus. Of note was that some known non-essential genes did not have any or only very few insertions in this approach. This observation was explained by dubiously annotated ORFs that overlapped with essential genes, by conditional essentiality or by the data analysis procedure in which repeated sequences were filtered from the reads. On the other hand, essential genes contained some transposons, specifically in permissive domains. The mutant libraries were also compared with each other to highlight that the differences in insertion locations were linked to medium usage or to the background strain/mutant (Michel et al., 2017).

*S. pombe* is another very popular model yeast, in which deletion collections exist (Kim et al., 2010). One of the first transposon experiments in *S. pombe* used Hermes to generate 360,513 insertions *in vivo* and in a naked gDNA control in order to perform an integration profiling study. The aim of the study was to identify genes essential for cell division. Only 33% of the transposon insertions occurred in the ORFs, and low integration densities corresponded to essential genes. There was, however, an exception to that: several mitochondrial genes, encoded in the nucleus, had high insertion densities even though they were believed to be essential. This is probably due to the existence of large protein pools, which segregate together with the mitochondria and were sufficient to sustain several generations without the presence of the encoding genes. Furthermore, some genes that were marked as non-essential inferred from deletion collection screenings were found to be essential by transposon insertion. These genes were later found mistakenly non-deleted in the deletion collection (Guo et al., 2013). Both of these experiments show that transposon insertion mutagenesis libraries contain even more information compared to classical deletion collections.

Several yeast species are used in industry in order to produce specific molecules at high quantities. *K. phaffii*, previously classified as *Pichia pastoris*, is the most popular yeast system for expression of recombinant proteins (Schwarzhans et al., 2017). Its genome sequence is known, yet most genes had not been functionally annotated until recently. Two different transposons have been used in *K. phaffii* to determine gene essentiality: TcBuster and Sleeping beauty. Both of them were used to construct several insertion libraries. Insertion-containing cells were enriched in selective liquid medium before sequencing. Sleeping beauty inserted in almost 800,00 unique sites, while TcBuster resulted in more than 130,000 independent insertions. Almost 40% of these insertions occurred in intergenic regions, which make up <20% of the genome. When insertions occurred in ORFs, they often positioned near the end of the gene. Insertion sites preferred by Sleeping beauty differed significantly from the ones preferred by TcBuster, Sleeping beauty inserted slightly more at the 3′-untranslated sequences of genes inserting almost randomly, while TcBuster preffered insertion in promoter regions. The different insertion sites led to a total of more than 200,000 unique insertion sites. Most genes with low insertion densities in these libraries were orthologs of essential genes in *S. cerevisiae* or *S. pombe*, validating the functionality of the libraries (Zhu et al., 2018a).

*Yarrowia lipolytica* is an oleagineous yeast, which is often used for the production of hydrocarbon-based chemicals, as it can accumulate up to 90% of its cellular weight in lipids (Blazeck et al., 2014). For *Y. lipolytica*, only 25% of its genome features are presently functionally annotated, for many others their annotation is strictly based upon homology with *S. cerevisiae* or *S. pombe* (Magnan et al., 2016). The *Hermes* transposon was used to generate 534,000 independent insertions. This led to the classification of roughly 22% of the genes as essential, most of which were homologous to *S. cerevisiae* and *S. pombe* essential genes. More respiratory genes are essential in *Y. lipolytica* compared to the two model species. Most hits were intergenic, with a frequency of one hit every 24.37-bp. For intragenic insertions the frequency was about one hit every 97.5-bp. Insertion mutants were grown in competition over several generations, which showed that more stringent/competitive environments led to a lower variability in the mutants. For example, fewer independent insertion mutants were present after growth on glycerol compared to glucose. Furthermore, the insertion library was screened for accumulation of lipids and several mutants were identified. All the mutants exhibited insertions in intergenic regions, mostly between two divergent genes. This means that the insertion possibly affected not just one, but two adjacent genes (Patterson et al., 2018).

Yeasts can also negatively impact our daily lives, as is the case for pathogenic organisms. *C. albicans* is one of the most common human fungal pathogens. This fungus is present as a commensal in a significant part of the population and can cause severe invasive infections in immunocompromised individuals. Next to that, superficial infections, mainly on the oral or vaginal mucosa are quite common, even in immunocompetent people (Kim and Sudbery, 2011). Only very recently transposon mutagenesis was used in order to investigate gene essentiality and drug resistance in this fungus (Gao et al., 2018; Segal et al., 2018). The construction of transposon insertion mutants in this fungus was long thought to be compromised by its diploidy and lack of meiosis. In 2013, however, it was discovered that haploids could be obtained through concerted chromosome loss. These haploids were unstable and grew significantly slower than related diploids (Hickman et al., 2013). A derived haploid strain was described, that was selected for its faster growth and, as such, could be used for the construction of a transposon mutant library. The *Ds* element from maize, containing a selective marker for nourseothricin, was inserted into the 5'-region of *ADE2*, thus inactivating this gene. A hyperactive transposase was expressed from an inducible promoter, which effectively induced excision and insertion of the transposon (Mielich et al., 2018). This system was used to construct a library from which more than 500,000 independent insertion sites were mapped, with one third of them occurring in annotated features in the *C. albicans* genome. Around 95% of all annotated features contained at least one transposon insertion. This library was then used to determine gene essentiality under standard laboratory conditions, since essential genes represent potential drug targets. More than 1,500 genes were identified as essential, most of which were essential in *S. cerevisiae* and/or *S. pombe*. However, some non-essential orthologs of *S. cerevisiae* or *S. pombe* seemed to be essential in *C. albicans*. This is probably due to functional redundancy due to duplications and paralogs in the model yeasts. Some *C*. *albicans* essential genes did not have orthologs in either of the model yeasts and are poorly characterized. They could serve as specific drug targets, especially since some of them have no human homologs and are conserved in other fungal pathogens such as *Aspergillus fumigatus* and *Cryptococcus neoformans* (Segal et al., 2018). *C. albicans* haploids were also used for piggyBac transposition. An insertion frequency of about 5% was reached, leading to insertions in all chromosomes in nearly 5,000 genes. Genes without transposon insertions were probably essential (Gao et al., 2018).

Infections with a second *Candida* species, *C. glabrata* are increasing, especially in the western world. This haploid yeast is considered less virulent compared to *C. albicans*, yet rapidly acquires resistance to the most used antifungal fluconazole (Dyer and Paoletti, 2005). A Hermes transposon was used in a clinical isolate of *C. glabrata*, generating more than 500,000 independent insertions. These were used to identify essential genes. About 84% of essential genes in *C. glabrata* had orthologs that are essential in *S. cerevisiae*. Interestingly, genes from the spliceosome complex, which are essential in *S. cerevisiae*, were non-essential in *C. glabrata*. This could be explained by the lower presence of introns in *C. glabrata* compared to baker's yeast. On the other hand, genes involved in polyamine biosynthesis were essential in *C. glabrata* but not *S. cerevisiae* under the used growth conditions (Gale et al., 2020). In a separate study, Levitan et al. (2020) compared three different transposons in three different yeast species for their ability to predict gene essentiality. Data from previously published AcDs, Hermes and piggyBac transposon studies in *S. cerevisiae, S. pombe* and *C. albicans* were used in a machine learning approach (Li et al., 2011; Guo et al., 2013; Michel et al., 2017; Gao et al., 2018; Segal et al., 2018). Interestingly, the authors found that a library with many independent insertions would significantly improve performance, while increasing reads per insertion did not have a similarly strong effect. Gene essentiality predictions using the piggyBac-data in both *S. pombe* and C. *albicans* were the least efficient, since the specific target site, TTAA, is not present in all ORFs throughout the fungal genome. This also led to a lower transferability of the piggyBac data compared to Hermes or MiniDs data. This means the machine learning algorithm can be trained with a MiniDs dataset in order to analyze Hermes data, but not piggyBac data. Furthermore, the AcDs transposon exhibited cryptic promoter-enhancer activity, which can result in increased expression of downstream ORFs when inserted in promoter sequences. This leads to *S. cerevisiae* essential genes tolerating MiniDs insertion, yet not Hermes insertion. Interestingly the authors found that essential genes were species- and condition-specific (Levitan et al., 2020).

It is often interesting to identify genes which are necessary for growth in specific conditions, as this can create a more in-depth understanding of pathways needed to metabolize specific nutrients for example. Since *K. phaffii* is known for its ability to grow on methanol (De Schutter et al., 2009), the libraries made by TcBuster and Sleeping beauty mentioned before, were also used to screen for loss of growth on methanol. More than 100 genes with a possible function in methanol metabolism were thus identified, some of which were previously known to be involved in this process (Zhu et al., 2018a). Another method to identify genes necessary for growth on methanol in *K. phaffii*, is the use of 2-deoxyglucose (2-DG), non-metabolizable a glucose analog. Upon growth on 2-DG and methanol, only cells that can use methanol as a carbon source can grow. A piggyBac library was screened for genes involved in resistance to 2-deoxyglucose (2-DG) The authors identified five genes involved in 2-DG resistance, including *SNF3, GRR1*, and *MIG1*. Some of these genes had been linked to methanol metabolism previously, which confirmed the validity of the system (Hoepfner et al., 2014; VanHoute and Maxwell, 2014).

### Investigating Genetic Interactions

Specific mutations can be an advantage or a disadvantage for a yeast, or even both at the same time. A deletion that results in slower growth, yet better tolerance to a certain stressor, can help in processes in industry. Ideally, it would be possible to find additional modifications that stabilize the tolerance effect, meanwhile rescuing the growth defect. Transposon mutagenesis can be used to identify such genetic interactions (Li et al., 2011; Yusa et al., 2011; Michel et al., 2017; Edskes et al., 2018). Furthermore, large screening for genetic interactions can help establish networks of functional connections between genes and pathways. Transposon insertion mutagenesis can be used both for extensive screening, when the whole library is sequenced as well as for identifying specific mutations, by only sequencing the mutants that show the desired phenotype. The possibility to only sequence specific mutants makes the process cheaper and easier, as only few sequencing reactions are needed.

In the saturated transposon mutagenesis work of Michel et al. (2017) in *S. cerevisiae*, two proof-of-principle genetic interaction experiments were included. In one of them a transposon insertion library was made in strains containing a combination of a mutation in the ER-mitochondria encounter structure gene, *MMM1*, and its suppressor mutation in endosomal protein Vps13. Previously identified genes, that are necessary for proper functioning of this suppressor mutation, indeed became essential in this insertion library, even if they were not essential in the insertion library created in the wild type strain. In a second experiment, two libraries were created including one in a *DPL1*-deletion strain and the other in a *dpl1/psd2* double deletion strain. These genes were previously demonstrated to function in phosphatidylethanolamine biosynthesis. In the double deletion strain, phosphatidylethanolamine can only be generated in the mitochondria by means of Psd1, so that *PSD1* as well as genes for lipid shuttling to and from the mitochondria will be essential. This was confirmed using transposon insertion mutagenesis (Michel et al., 2017).

Hermes transposition was also used to identify genes that could rescue a mutant phenotype, more specifically the growth defect induced by the *URE-3* prion. A comparison of mutagenesis libraries between a strain that was under constant selection for the presence of the prion and a control strain, resulted in genes with insertions in the control, but not in the prion-containing strain. It was shown that a mutation of *GLN1*, encoding glutamine synthetase, or a previously uncharacterized gene, *LUG1*, could rescue the growth defect (Edskes et al., 2018).

Since piggyBac is currently the most commonly used transposon system, it was also used in *S. cerevisiae*. The system was used to identify mutations rescuing the cell wall defects of a mutant in the *OCH1* gene, which is used in glyco-engineered yeast cells for expression of human-compatible glycoproteins. The deletion eliminates yeast specific N-glycan structure production. One mutant was found with the ability to rescue the growth defect of the parental strain. It was proven that this phenotype was linked to transposon insertion, since removal of the element resulted in the parental defect. Further demonstration for the validity of the approach came from the nature of the investigated gene: the insertion occurred in the *BEM4* promoter, which resulted in the upregulation of the downstream gene. Bem4 belongs to the same pathway as Rho1, a protein already known to be involved in rescue of the *och1* phenotype (Mutumwinka et al., 2018).

The piggyBac transposon system was used in *S. pombe* to identify mutants that suppress the temperature sensitive growth defect of a *cdc25-22* strain. All mutants that rescued the phenotype had insertions in the *WEE1* gene, which had previously been identified using chemical mutagenesis (Li et al., 2011).

### Drug Target Identification

Several yeast species have negative effects either on our own bodies or on the production of our food. Antifungal drugs are used commonly in the clinic as well as the agricultural sector. Only few classes of antifungals are available and resistance to all of them is increasingly present. Therefore, it is of high importance to develop new drugs as well as to identify resistance mechanisms, as this can help to develop synergistic therapies.

In their extensive *S. cerevisiae* functional mapping study, Michel et al. (2017) also included a screening for targets of the drug rapamycin, which blocks cell proliferation. They showed interruption of several genes, among which *FPR1*, the rapamycin receptor, and other genes known to be involved in rapamycin signaling (Michel et al., 2017).

In *S. pombe*, the pre-constructed library using piggyBac was applied to screen for mutants resistant to thiabendazole, a drug affecting microtubules (https://pubchem.ncbi.nlm.nih.gov/compound/Thiabendazole). Seventy-three resistant mutants were isolated, revealing a novel gene involved in thiabendazole resistance, *DAM1*, a gene required for proper chromosome segregation. The insertion mutant in *DAM1* caused a gain-of-function (thiabendazole resistance). It is therefore always important to verify whether or not specific transposon insertions cause a gain- or a loss-of-function. Interestingly, Li et al. (2011) found in this experiment several resistant mutants that were not caused by transposon insertion, but simply by spontaneous mutation. It is therefore important to verify whether removal of the transposon reverts the original phenotype (Li et al., 2011).

In the clinic, fluconazole is one of the most commonly used drugs to treat fungal infections. Unfortunately, resistance to this drug is becoming increasingly widespread. Gao et al. (2018) used piggyBac transposition in *C. albicans* haploids in order to screen for genes involved in fluconazole resistance. This led to the identification of several genes that improve fitness during growth in the presence of the drug. Several of them were already known to be involved in this process including members of the ergosterol biosynthesis pathway and its regulation including *ERG3, ERG6*, and *ERG251*. Additionally, identified genes were linked to ergosterol and sphingolipid biosynthesis. Upon further characterization of genes involved in sphingolipid biosynthesis (*FEN1* and *FEN12*), a novel *in vitro* resistance mechanism to fluconazole was discovered (Gao et al., 2018).

Furthermore, the transposon insertion library created with Hermes in *C. glabrata* was also used to identify genes that regulate susceptibility to fluconazole. Several genes that were previously linked to fluconazole resistance, were identified in this approach, validating the use of insertion profiling for this purpose. On the other hand, several genes that were not linked to fluconazole resistance up to now were identified, among them α-ketoglutarate metabolic genes (*KGD1, KGD2, IDH1*, and *IDH2*). Their role in fluconazole susceptibility was confirmed using deletion mutants (Gale et al., 2020).

### Essential Protein Domain Identification

During their saturated transposon mutagenesis in *S. cerevisiae*, Michel et al. (2017) noticed that it was not only possible to identify essential genes, but also essential protein domains. As multiple insertions can be present in the same gene, attention should be paid to the position of transposon insertions within the coding sequence. An example is *GAL10*, which encodes a bifunctional enzyme with epimerase and mutarotase activities. In the experiment, cells were grown on a mixture of α- and β-galactose to avoid the need for conversion of one form into the other. The mutarotase domain exhibited several insertions in the transposon library and facilitated the identification of domains essential for its activity (Michel et al., 2017). During the aforementioned rapamycin screen insertions were found in *PIB2*. This was unexpected, since *pib2* deletions are known to be sensitive to rapamycin. A more in-depth look showed that insertions were only present at the N-terminal end of the encoded protein and resulted in a gain-of function effect on *PIB2*, as such explaining rapamycin resistance (Michel et al., 2017).

## Perspectives

The use of heterologous transposition in fungi has been investigated for over 10 years, however, the creation of extensive insertion libraries is a more recent development. Due to the availability and affordability of fast sequencing platforms, it is now much easier to map and identify mutants in these libraries. So far this strategy has been mainly used in model yeasts and industrially important fungi. The need for only one round of transformation, an often difficult ordeal in non-model yeasts or specific industrial, medical or environmental strains, makes transposon mutagenesis a fast and effective way to create mutant libraries covering entire genomes. Especially in fungi where no deletion collections are available, transposition mutagenesis could facilitate research significantly. Furthermore, most transposons insert both in intergenic regions, as well as in ORFs, and thus yield alternative information that deletion collections cannot deliver. An insertion in a regulatory region of a gene can influence a phenotype in a different way compared to full deletion. One disadvantage of this technique is that the transposon insertion sites in a given library will always be constrained to the conditions prevailing during its creation. In addition, since selection for transposon insertion is needed, genes that are needed for this selection will always be essential in the library, even though they might not be essential in other conditions.

There is still a huge possibility for the use of transposon libraries, mainly in plant and animal pathogenic fungi. The main advantage is that once libraries are made, they can be stored and reused for tests in other environments or in the presence of drugs, as well as the possibility to use plasmids in other background strains or mutants of the same species. For example, Jorgensen et al. (2020) used the plasmids of the extensive functional mapping study in *S. cerevisiae* by Michel et al. (2017) in order to investigate how a strain defective in ER-plasma membrane tethering adapts to the loss of these contact sites (Jorgensen et al., 2020). Meanwhile Haribowo et al. (2019) used the same plasmids in a deletion strain that is sensitive to the 1-deoxysphinanine (DoxSa) in order to gain understanding of the cytotoxicity of deoxysphingolipids and related molecules (Haribowo et al., 2019).

A lot of attention has focused on how different background strains can react to specific stimuli in several organisms, among which *C. albicans* (Huang et al., 2019). Saturated transposon mutagenesis could be used for an in-depth comparison between background strains under defined conditions.

A particular environment that would be interesting to study for pathogenic organisms, is inside and/or on the host. This was recently successfully carried out for the fish infecting bacteria, *Edwardsiella piscida* during infection of fish. The Mariner transposon Himar1 was first used to generate a high-density *in vitro* library, which was then used to infect turbot intraperitoneally. Bacteria were recovered from the livers of infected fish at different timepoints for transposon quantification. It was then possible to determine the effect on fitness over time for each transposed gene (Yang et al., 2017).

Furthermore, there is a clear need to identify resistance mechanisms to commonly used drugs, in order to develop treatment strategies that will work in the clinic, even in the case of resistant organisms. Using a transposon mutant library, multiple resistance pathways might be identified, since many deleterious and regulatory mutants can be selected simultaneously in the presence of the drug. This has already been performed in reference strains of the human fungal pathogens *C. albicans* and *C. glabrata* (Gao et al., 2018; Gale et al., 2020). Yet it may be of particular interest to carry out these experiments directly in specific drug-resistant clinical isolates.

Important to note is that saturated transposon mutagenesis is not applicable for all fungi. There are still some basic requirements, which rule out several species. For example, species need to be cultured *in vitro* in the laboratory, which is not possible for specific fungi which are obligate symbionts, such as *Pneumocystis* spp. Furthermore, an efficient transformation method needs to be available in the species, since the plasmids/constructs containing the transposase and the transposon need to be added. Fortunately, there are still many industrial species, human pathogens and agriculturally interesting species in which all of these prerequisites are present and in which transposon mutagenesis could be used to acquire novel knowledge that could improve our lives significantly.

## Author Contributions

All authors listed have made a substantial, direct and intellectual contribution to the work, and approved it for publication.

## Conflict of Interest

The authors declare that the research was conducted in the absence of any commercial or financial relationships that could be construed as a potential conflict of interest.
